# 3D-Reconstruction of the human conventional outflow system by ribbon scanning confocal microscopy

**DOI:** 10.1371/journal.pone.0232833

**Published:** 2020-05-18

**Authors:** Ralitsa T. Loewen, Susannah Waxman, Chao Wang, Sarah Atta, Si Chen, Simon C. Watkins, Alan M. Watson, Nils A. Loewen

**Affiliations:** 1 Department of Ophthalmology, University of Würzburg, Würzburg, Germany; 2 Department of Ophthalmology, University of Pittsburgh, Pittsburgh, Pennsylvania, United States of America; 3 Department of Cellular Biology, Center for Biologic Imaging, University of Pittsburgh, Pittsburgh, Pennsylvania, United States of America; 4 Department of Ophthalmology, Xiangya Hospital, Central South University, Changsha, Hunan, China; Nicolaus Copernicus University, POLAND

## Abstract

**Purpose:**

The risk for glaucoma is driven by the microanatomy and function of the anterior segment. We performed a computation-intense, high-resolution, full-thickness ribbon-scanning confocal microscopy (RSCM) of the outflow tract of two human eyes. We hypothesized this would reveal important species differences when compared to existing data of porcine eyes, an animal that does not spontaneously develop glaucoma.

**Methods:**

After perfusing two human octogenarian eyes with lectin-fluorophore conjugate and optical clearance with benzyl alcohol benzyl benzoate (BABB), anterior segments were scanned by RSCM and reconstructed in 3D for whole-specimen rendering. Morphometric analyses of the outflow tract were performed for the trabecular meshwork (TM), limbal, and perilimbal outflow structures and compared to existing porcine data.

**Results:**

RSCM provided high-resolution data for IMARIS-based surface reconstruction of outflow tract structures in 3D. Different from porcine eyes with an abundance of highly interconnected, narrow, and short collector channels (CCs), human eyes demonstrated fewer CCs which had a 1.5x greater cross-sectional area (CSA) and 2.6x greater length. Proximal CC openings at the level of Schlemm’s canal (SC) had a 1.3x larger CSA than distal openings into the scleral vascular plexus (SVP). CCs were 10.2x smaller in volume than the receiving SVP vessels. Axenfeld loops, projections of the long ciliary nerve, were also visualized.

**Conclusion:**

In this high-resolution, volumetric RSCM analysis, human eyes had far fewer outflow tract vessels than porcine eyes. Human CCs spanned several clock-hours and were larger than in porcine eyes. These species differences may point to factors downstream of the TM that increase our vulnerability to glaucoma.

## Introduction

Recent experiments in ex vivo human [1] and pig [1,2] eyes confirmed an outflow resistance distal to the trabecular meshwork (TM) that can be reduced. In an anterior segment model, nitric oxide increased the outflow facility even after the TM was removed [2]. Spectral-domain optical coherence tomography (SD-OCT) could detect outflow tract vessel dilation at the level of the collector channels (CC) and scleral vascular plexus (SVP) [2]. The results of these recent studies match historic ones that examined the impact of nitroglycerin [3–5] and hydralazine [3] on intraocular pressure (IOP) and outflow. Distal outflow resistance is pronounced in glaucoma patients. TM bypass [6,7] and ablation [8–11] procedures were expected to reduce IOP to the level of episcleral venous pressure, around 8 mmHg [12]. Surprisingly, only approximately 0.3% of surgeries achieve this goal [11]. Moreover, a correlation of pre- and early postoperative IOP (before glaucoma medications are resumed) suggests that the baseline IOP reflects the severity of post-TM outflow pathology [8]. A glaucomatous change of the distal outflow tract that can cause such a significant resistance in small vessels can likely only be detected by high-resolution imaging techniques.

In our porcine eye model, we recently refined a benzyl alcohol benzyl benzoate (BABB) protocol as a clearing technique to overcome the opaqueness of the sclera and apply high-resolution imaging to the deep perilimbal structures [13]. This requires staining the outflow vessels with lectin-labeled fluorophores and then using ribbon scanning confocal microscopy (RSCM) to reconstruct the outflow tract virtually. The process of assembling several million confocal images is computation-intense but could be accomplished using a high-performance computer cluster [13,14].

The human outflow tract has not been analyzed at such high resolution. Here, we apply this approach to two left eyes procured from octogenarian donors. The number of eyes had to be limited in this explorative study due to the demanding requirements for processing several terabytes of data. Canalogram studies of human eyes [15] suggested a perilimbal, proximal vessel network with a density similar to that of porcine eyes [16–19] which seemed to contrast our RSCM studies of this species [13]. Our RSCM studies in pig eyes found far more CCs than described for human eyes, with greater nasal CC openings, and a larger SVP in the inferior limbus than in other quadrants. Here, we hypothesized that an RSCM analysis of the lectin-labeled outflow tract of human eyes would reveal important species differences in a comparison.

## Materials and methods

### Whole eye lectin perfusion

No human subjects were involved in the investigation. Human donor eye specimens were received from the Center for Organ Recovery and Education (CORE, Pittsburgh, PA) that recovers organs from registered US donors. None of the donors were from a vulnerable population and all donors or next of kin provided written informed consent that was freely given to CORE for organ recovery to be used for transplantation or research. Importantly, research that involves the use of human specimens or data is not considered human subjects research if subjects are deceased [20]. Therefore, a consent specifically for the research conducted here by the donor or their next of kin was not required. Only whole eyes without a history of glaucoma that were procured within 48 hours of death were accepted and processed within two hours of arriving in the laboratory. We analyzed a left eye from an 85-year-old female (eye 1) and a left eye from an 88-year-old male (eye 2). Eye 1 had no ocular history and eye 2 had a history of phacoemulsification with a posterior chamber intraocular lens implant. The anterior chamber was cannulated with a 20-gauge needle positioned temporally and just anterior to the limbus. After perfusion with phosphate-buffered saline (PBS) for 30 minutes, fluid was drained to allow a bolus of 300 μl of a lectin-fluorophore conjugate (200 μg/mL), followed by constant-pressure infusion of the label at 20 μg/mL and 15 mmHg for 90 minutes as we have done before in porcine eyes [13]. Eyes were stained with rhodamine-labeled lectin (#RLK-2200, Vector Laboratories, Inc, Burlingame, CA, USA). The eyes were subsequently fixed by perfusion with 4% paraformaldehyde (PFA) for 90 minutes and left in 4% PFA overnight, bisected along the equator, anterior segments cut into quadrants, and marked with indicator cuts according to their anatomical position. The resulting anterior segments were then cleared with BABB according to prior protocols [13].

### Image capture

Samples were imaged and processed as described before [13,14]. Briefly, full-thickness perilimbal scans were acquired with a confocal microscope designed for high-speed ribbon-scanning and large scale image stitching (RS-G4, Caliber I.D., Andover, MA, USA). The system was fitted with a scanning stage (SCANplus IM 120 × 80, #00-24-579-0000; Märzhäuser Wetzlar GmbH & Co. KG, Wetzlar, Germany) and an Olympus 25×, 1.05 NA water immersion objective (XLPLN25XWMP2; Olympus). Volumetric scans were acquired with a voxel size of 0.365 × 0.365 × 2.43 μm. A scan-zoom of 1.5 was used during acquisition to achieve the desired resolution. Images were acquired over a single channel with an excitation wavelength of 561 nm and an emission filter of 630/60. Laser percentage, high voltage (HV), and offset were held constant throughout the volume at 7, 85, and 15, respectively.

### Image processing

The resulting series of Tagged Image File Format (TIFF) volumes comprised of 422 to 520 images were converted to the Bitplane Imaris IMS format. Each of the volumes contained between 548 and 889 gigabytes of data. To facilitate analysis, each of the IMS files was placed on a specialized high-speed solid-state file server equipped with 20 gigabits of network bandwidth. The IMS files were made accessible to multiple workstations equipped with a recent high-end Intel i7 processor and an NVIDIA GTX 1080 graphics card. All workstations were connected by 10-gigabit networking. We reconstructed the limbal outflow structures with image analysis software (Imaris 9.2, BitPlane AG, Zurich, Switzerland) and explored it in both 2D and 3D views. Green was the default setting for a detected fluorophore in this imaging program and represented binding of the label to lectin binding glycocalyx. We identified the TM, Schlemm’s canal (SC), CCs, and SVPs with the surface function in Imaris. The range of grain size values used to resolve these structures was between 2.6 to 3.0 μm. Other parameters were a background subtraction with a diameter of the largest sphere fitting into the object of 10 to 200 μm, a background subtraction threshold value of 480 to 1387, and a filter to remove particles under 1.0×104 to 4.2×108 voxels. We created representative surfaces of the outflow tract structures up to 1000 μm from the distal TM. Surface creation parameters were refined to optimally resolve the structural details of each region. Detailed Imaris surface creation parameters are provided in S1 Table. These surfaces were manually labeled as either TM/SC, CC, or SVP.

The TM was defined by a densely stained region of porous tissue at the base of each quadrant that appeared together with distinct beams. The beginnings of the CCs were defined by the appearance of a dark lumen surrounded by a bright wall with a beginning at the level of SC and a distal connection to the relatively superficial, perpendicularly oriented SVP. We observed different types of CC patterns of reaches at their catchment scale: meandering, braided, anabranching [21], and anastomosing. Given these differences, we decided to introduce the term “CC unit” as a region with a singular catchment area.

Using Imaris slice mode, we counted and measured the CC openings along the XY-plane at their sites of connection to the SC and SVP. The widest and narrowest lengths of these openings were recorded. All measurements were taken at the outermost point of proximal and distal confluence. Outlier CCs that continued beyond the 1,000 μm mark were reconstructed for visual representation.

Outlines of the projections of long ciliary nerves, known as Axenfeld loops [22–24], could readily be seen before clearing and were later identified in Imaris as dark structures not labeled with lectins surrounded by microvessels resembling the perineurium. They were followed through slice mode to determine their path. We inverted the brightness values to highlight structures not labeled with lectins.

### Statistical analysis

The parameters of volume and location were obtained for TM and SC, CC, and SVP with the Imaris statistics function. CC opening lengths and widths were measured in Imaris slice mode. Location, cross-sectional area (CSA), and ellipticity for all CC openings were recorded. Data were analyzed by location for each eye. Data from human eyes in this study were compared to porcine eyes from our prior study [13]. Statistical tests were performed in Python 3.6.

## Results

**Fig 1:**
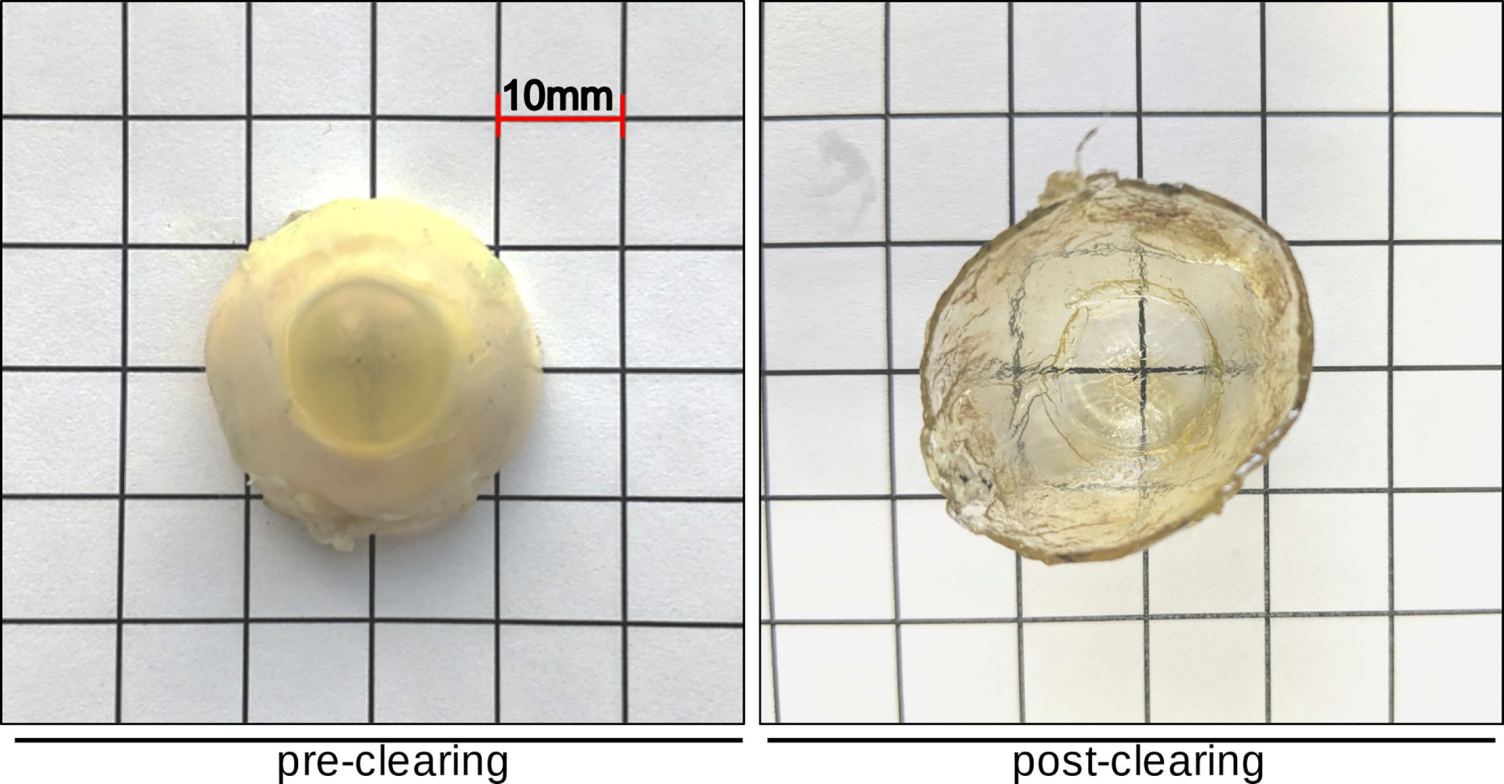
Macroscopic view of a BABB-cleared human eye. Human anterior segment pre and post BABB clearing.

**Fig 2:**
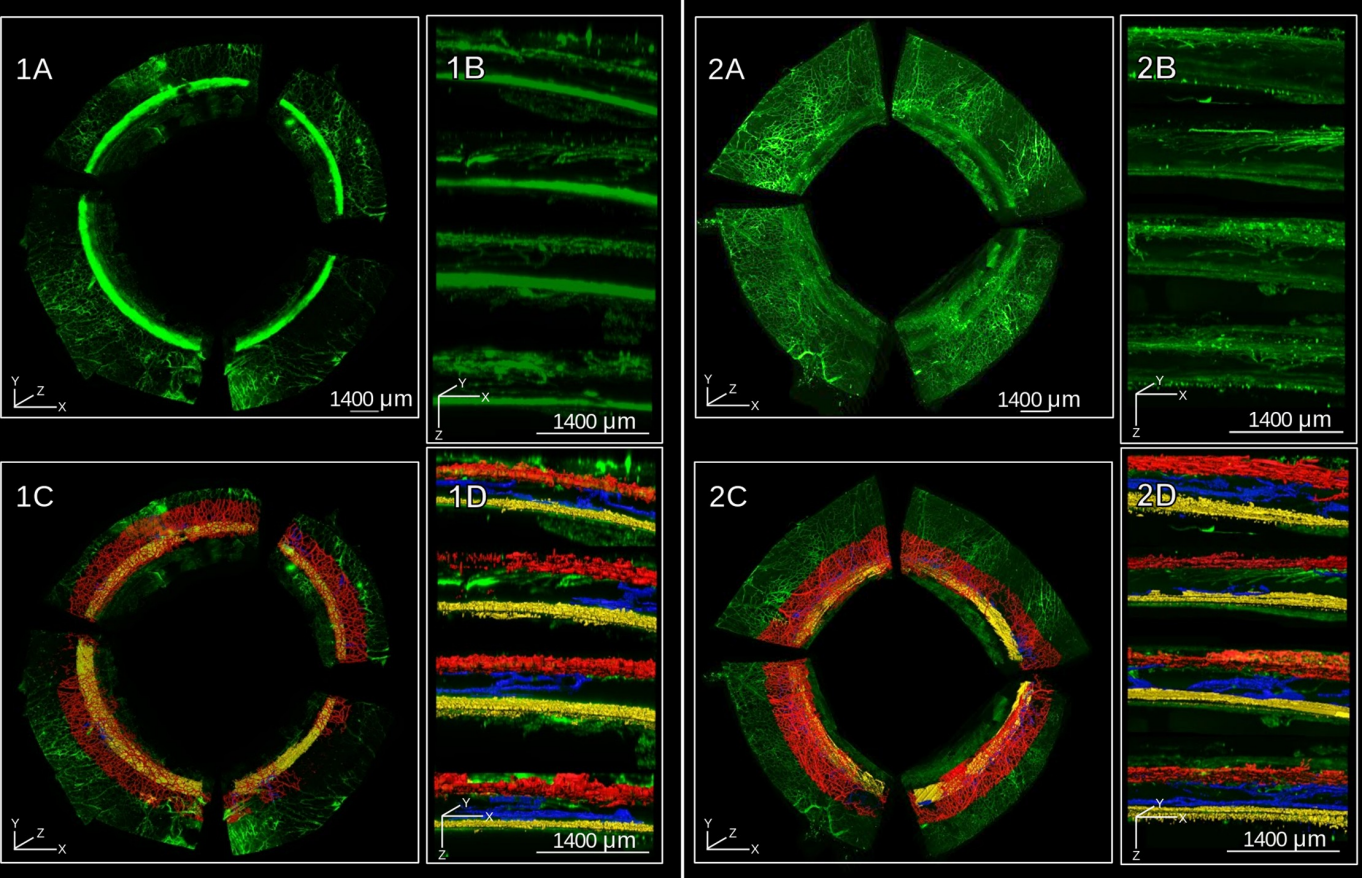
Volumetric limbal reconstruction by ribbon scanning confocal microscopy. RSCM reconstruction of eye 1 (1) and eye 2 (2) lectin-fluorophore perfused human eyes. Frontal (A) and sagittal (B) view of volumetric RSCM reconstructions. Frontal and sagittal views of labeled outflow tracts surfaces (C and D, respectively) with TM/SC in yellow, CC in blue, and SVP in red.

Whole human eyes could be labeled and cleared in approximately seven days using our modified BABB protocol [13]. Fig 1 shows an example of how the normally opaque sclera becomes transparent (pigmented structures consisting of the uvea, ciliary body, and iris, were removed **(Fig 1)**). Unlike other species, human sclera mostly lacks pigmentation and became completely transparent when the refractive index of the normally opaque tissue matched that of BABB. This occurred during the final BABB step and took about 20 minutes. The eyes could be left for as long as 2 months in BABB without a notable difference in transparency or fluorescent signal. The scan time was 64 hours for eye 1 and 51 for eye 2, respectively. Lectin-labeled cleared anterior segments could be imaged via RSCM **(Fig 2)**. Lectin-labeled fluorophores intensely stained the entire outflow tract from the TM to the SVP. In eye 1, the SC was easy to delineate from the TM. However, SC in eye 2 could not be delineated from the TM as in eye 1. The conversion time from TIFF images to Imaris files was 6 hours for eye 1 and 8 hours for eye 2. The lectin binding pattern matched the one observed in our prior studies [13].

**Fig 3:**
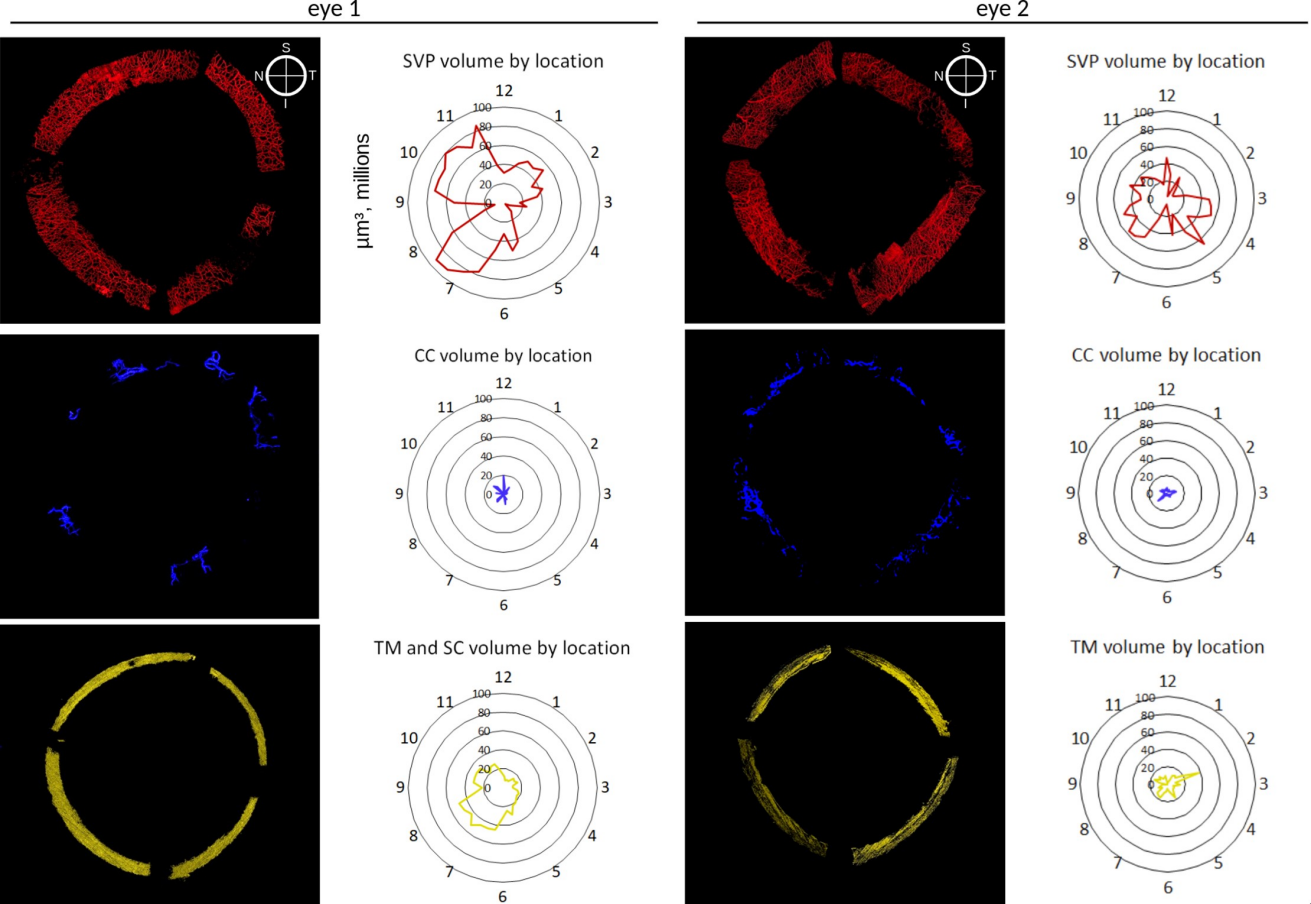
Radial charts with volumes. Frontal view of outflow tract structures. Volume measurements are plotted to correspond to their anatomical locations. TM/SC in yellow, CC in blue, SVP in red.

**Fig 4:**
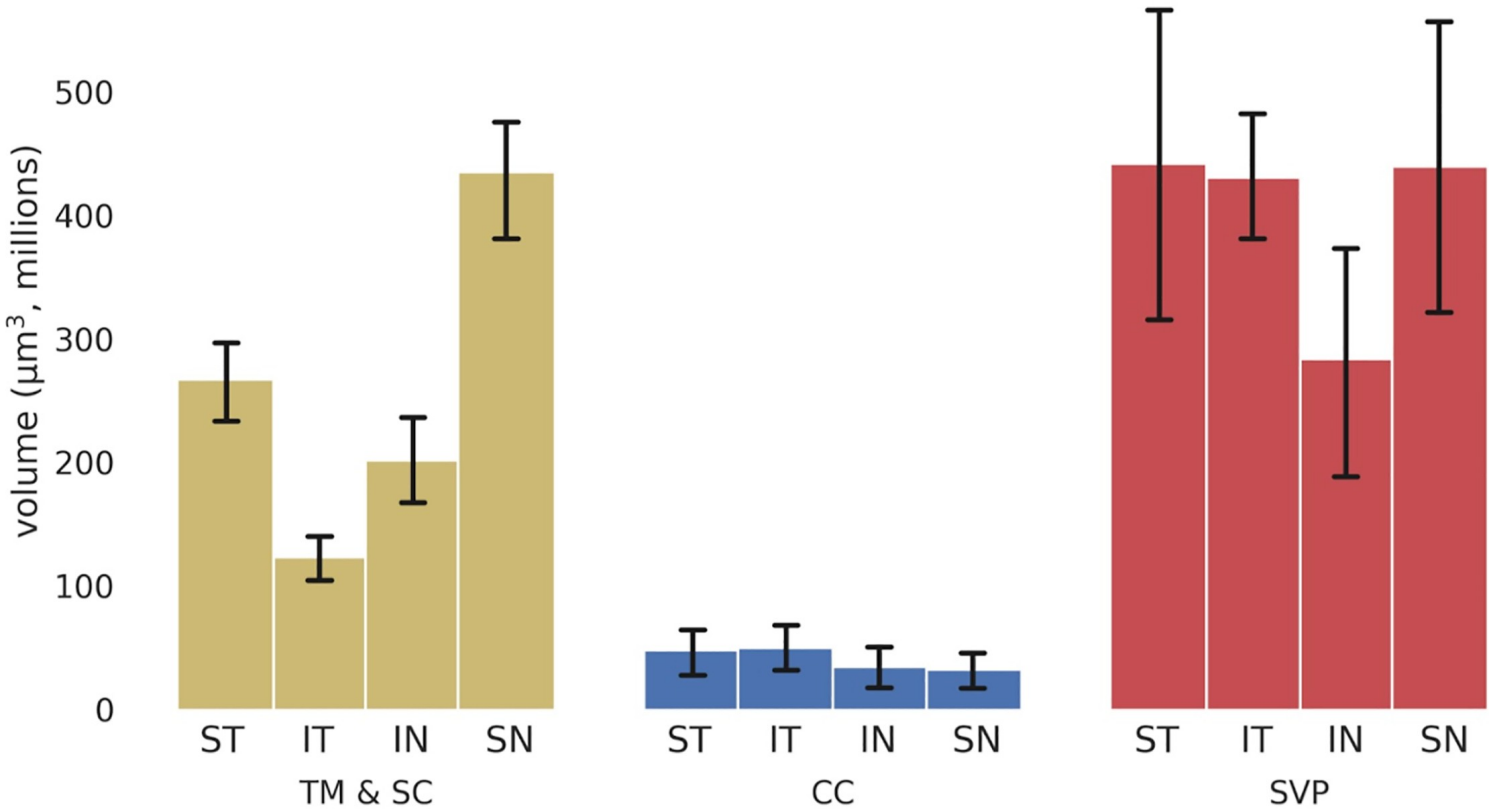
Outflow tract volumes. Volumes of outflow structures by anatomic location for eye 1 (top) and eye 2 (bottom) eye. SN: supranasal, ST: supratemporal, IT: infratemporal, IN: infranasal (averages with error bars using standard deviation).

**Fig 5:**
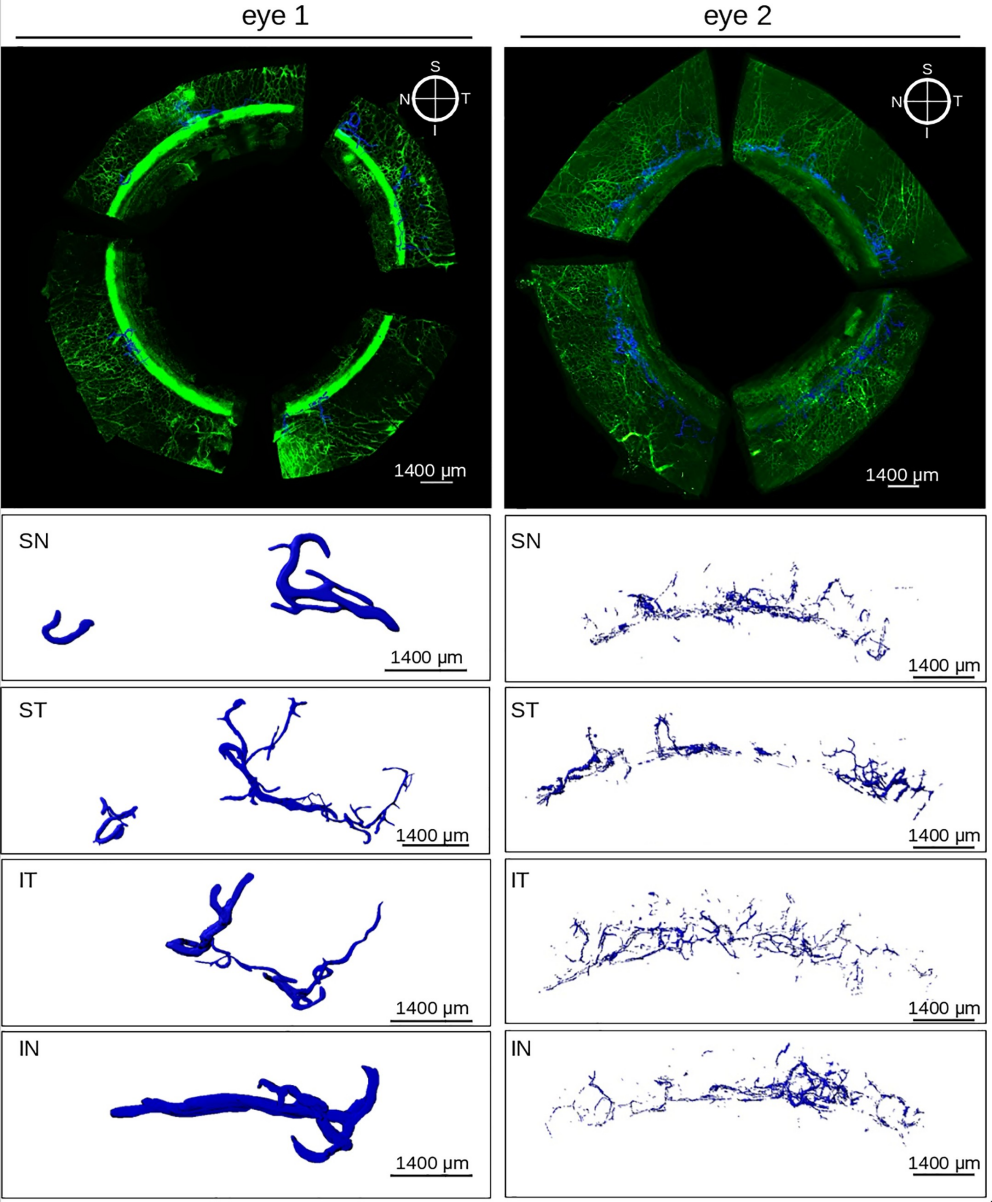
Collector channel morphology. Top panels show a volumetric reconstruction of eyes with CCs in blue. Bottom panels SN-IN show magnified CC morphology in each quadrant. SN: supranasal, ST: supratemporal, IT: infratemporal, IN: infranasal.

Computed volumes were displayed as radial charts **(Fig 3**) according to the anatomic location in frontal view. Volume and region-dependent differences are presented in **Fig 4**. The supranasal quadrant (SN_q_) of eye 1 had the largest SVP volume. The largest volume of TM and SC in this eye was in the infranasal quadrant (IN_q_). In eye 2, the largest SVP volume was in the infratemporal quadrant (IT_q_). The largest TM and SC volume in this eye was in the supratemporal quadrant (ST_q_). Some CC units had a wider area of catchment compared to others. Most of the CC units initiated from SC via several individual collectors before converging into a single lumen. In eye 1, 13 proximal CC openings connected to 20 distal CC openings through eight CC units. In eye 2, 13 proximal CC openings connected to 15 distal CC openings through 18 CC units. CC volume was greatest in the ST_q_. The lowest CC volume was found in the IN_q_ of eye 1 and the IT_q_ of eye 2. In eye 1, CC volumes were about 13.3 times smaller than the SVP volumes, while in eye 2, CC volumes were 7.7 times smaller than SVP volumes. There were strikingly different CC morphologies that varied from quadrant to quadrant **(Fig 5)**.

**Fig 6:**
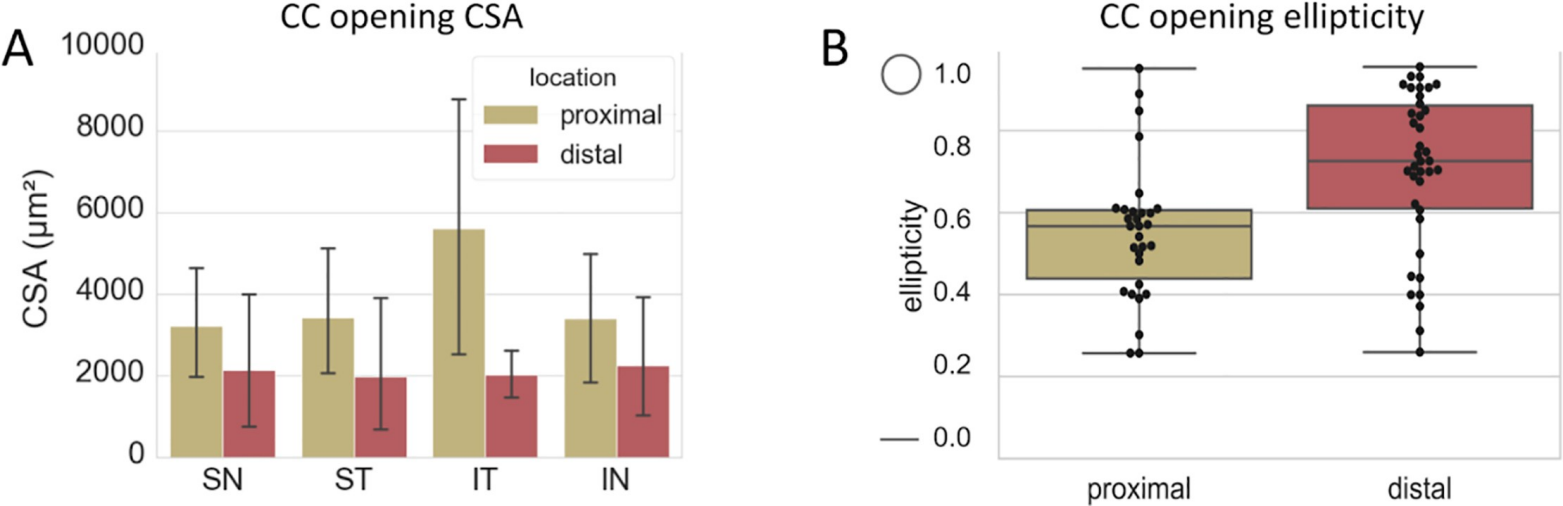
Cross-section areas and ellipticities of CC orifices at their proximal points of origin and at their distal points of connection with the SVP. A) Cross-section areas (CSA) of CC openings at their proximal points of connection with SC and their distal points of connection with the SVP. SN: supranasal, ST: supratemporal, IT: infratemporal, IN: infranasal. B) CC opening ellipticities of the proximal and distal ends. 1.0: perfect circle, 0.0: perfect line (averages with error bars using standard deviation).

There were segmental differences in the CSAs of CC orifices at their proximal openings at the level of SC and at their distal points of confluence with the SVP **(Fig 6A)**. CC opening CSAs ranged from 9911.1μm^2^ to only 161.5μm^2^. Proximal openings were significantly larger than distal ones (P = 0.017, 71.6%). The largest difference in average proximal and distal CSA was in the IT_q_ and the least difference in the ST_q_. The human CC opening CSA was significantly larger than the porcine CC opening CSA (p = 7.37×10^−9^). The ellipticity of CC openings was compared at their proximal and distal ends. CC openings were more circular distally than proximally (1.42 and 1.18 times, respectively, P = 0.0009) **(Fig 6B).** There was a significant linear relationship between CSA and cross-section circularity in both the human (P = 1.88×10^−6^, r = -0.46) and porcine eye examined before [13] (P = 0.0018, r = -0.21, linear regression) indicating that a large CSA is correlated with higher ellipticity or flatness.

The average CSA of individual CCs along their complete lengths in the human eye was 5221.1± 3521.1 μm^2^, greater than those in the porcine eye, 3374.0±2472.8 μm^2^ (P = 0.002, 1.54 times greater, **S1 Fig**). However, there was a lower CC density (0.52% coverage) in the human eye compared to the porcine eye (1.00% coverage, 1.92 times greater, P = 0.014). In a representative sample of CC lengths measured, human CCs traveled 2533.4±1413.6 μm, a significantly longer distance than traveled by those in the porcine eye, 994.0±194.5 μm (P = 0.004, 2.6 times longer, **S1 Fig**). The elements of the conventional outflow system were assembled into a flythrough movie of eye 1 with all quadrants **(S1 Movie)** and at higher magnification the supranasal quadrant **(S2 Movie)**. The complex physical relationship between CC units, proximal and distal CC opening, and SVP could be best appreciated when viewed as a movie **(S1 and S2 Movies).**

**Fig 7:**
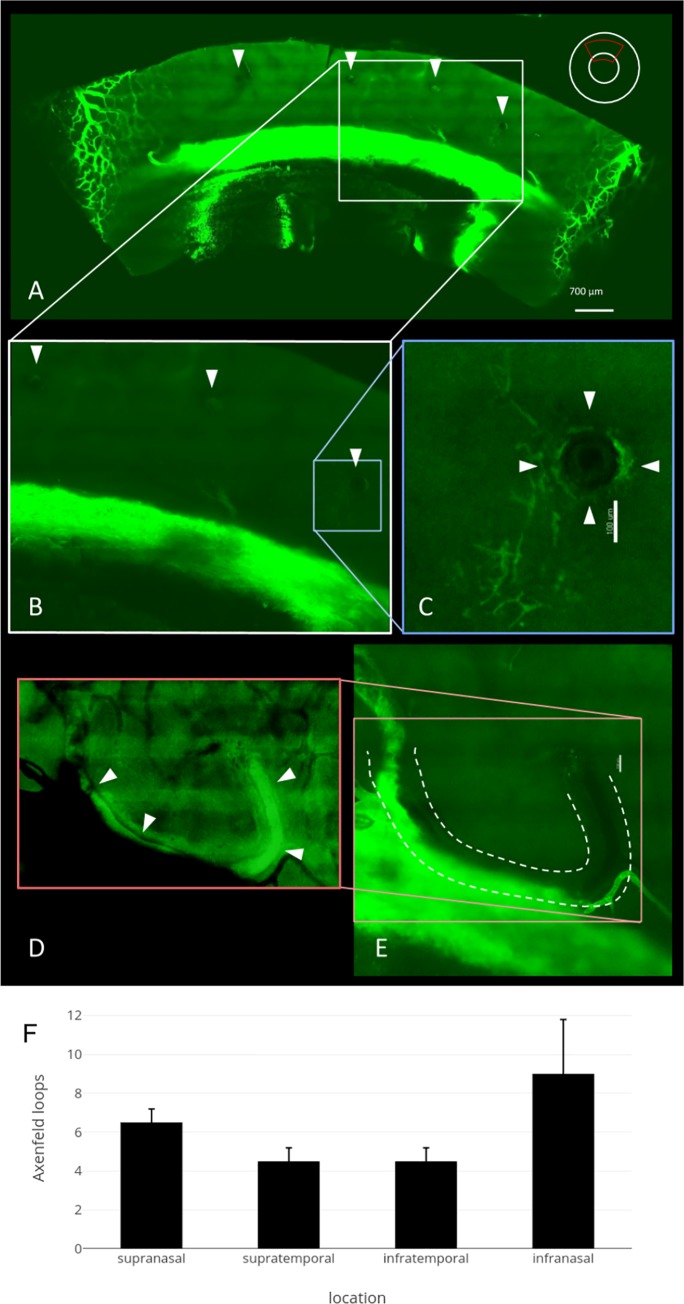
Axenfeld nerve loops could readily be identified in all quadrants. A) Upper quadrant of eye 2. Axenfeld loops are visible as dark structures void of lectin staining (white arrowheads). B) Magnified view of the area in A shows Axenfeld loop that enters the sclera. C) Magnified view of Axenfeld loop (white arrowheads) with perineural vasculature. D) The intrascleral path of the long ciliary nerve with Axenfeld loops can be more easily appreciated by image inversion. E) Direct proximity to the outer wall of Schlemm’s canal. F) Axenfeld loops per quadrant (averages with error bars using standard deviation).

In eye 1, 27 structures resembled Axenfeld loops with an average of 6.8±2.3 per quadrant. In eye 2, 22 such loops were seen with an average of 5.5±1.0 per quadrant. Most could be found in the IN_q_ followed by the SN_q_ **(Fig 7)**.

## Discussion

A growing body of evidence points to an outflow resistance distal to the TM as an essential aspect of both healthy [3,25,26] and glaucomatous [8–11] physiology. In this study, we provided a comprehensive overview of the human conventional outflow tract’s 3D architecture at an unprecedented resolution. We analyzed variations in the microanatomy and catchment of CC units. We assessed the similarities and differences we found between the human and porcine outflow tract. Fluorophore-labeled lectins were able to stain the human outflow tract, validating the modified BABB protocol we developed in porcine eyes for imaging the limbus full-thickness at submicron confocal microscopy resolution [13]. Lectins are carbohydrate-binding proteins with a high specificity [27,28] and can be used to study vessels by binding to their glycocalyx [29–31]. This new tool might be especially useful as Sienkiewicz et al.’s data [32] suggests that the accumulation of glycosylation end products in the TM may play a role in glaucoma. Although the current RSCM data acquisitions created 4-fold more data compared to our prior study, the process from staining to clearing and scanning was reasonably fast, taking approximately one week per eye, whereas a conventional confocal microscope would have required many months for the same acquisition. The combination of specialized toolsets to facilitate analysis, including purpose-built solid-state file servers and the Bitplane Imaris software suite, were critical to improving handling. These methods provide a new tool to researchers working on deciphering the aspects of structure and function of the outflow tract at high 3D resolution.

We observed significant inter-quadrant and inter-individual variations in the microanatomy of the outflow tract through volumetric reconstruction of RSCM-derived images. Our findings match Bentley et al. who found that proximal CC openings varied from simple and ellipsoid to tethered flaps and bridges [33]. In the future, fluid dynamics studies that use RSCM data-based mesh surfaces will examine the specific roles and impacts of these different CC unit morphologies.

The importance and potential vulnerability of CCs are highlighted by the fact that their total volume is only about 10% of the volume of the SVP. Depending on the compliance of CCs and the surrounding tissue, it is possible that the highly elliptical shape of larger CSA vessels may imply that the tract’s capacity for fluid transport has not been reached. With higher local pressures, elliptical cross-sections may be forced into a fully turgid circular shape, as already seen in the smaller distal CC openings, to act like pressure valves that maintain IOP at a set minimum value.

The number of structures we termed collector channels differs from other publications. While we observed 13 openings towards Schlemm’s canal, these collector channels had 18 to 20 distal openings. This is similar to Rohen and Rentsch, who observed 15 to 20 structures draining into episcleral veins [34]. In contrast, Hann et al. counted 24 to 29 [35] using micro-CT with a resolution of 2 to 5 micron. Although our XY-resolution was 5 to 14 times higher at 365 nm, the main differences may occur when intricately entangled 3D objects are counted.

The structural relationship of CC units, proximal and distal CC opening, and SVP is somewhat difficult to envision due to the complexity of the physical relationships and the lack of standardized terminology for it. This can perhaps be best appreciated in frame 4095–4291, time 3:24–29 of movie 1 (S1 Movie) by providing a useful visual tool. The intrascleral channels oriented parallel to Schlemm's canal are likely of particular relevance to the pathogenesis of glaucoma and to the cause of success or failure of microincisional glaucoma surgery that removes or bypasses the TM [36].

While the RSCM analysis of BABB-cleared porcine eyes demonstrated CCs that were numerous, short and direct [13], we found that CCs in human eyes were only about half the number of porcine eyes and traveled over twice the distance from Schlemm’s canal to the SVP. These results indicate that human eyes not only have far fewer collector channels but that their anatomy differs fundamentally by spanning several clock hours and having a 50% greater CSA. This came as a surprise because recent lower resolution canalogram studies of perilimbal outflow structures suggested a density and function of outflow vessels in human eyes [15] similar to that in porcine eyes [16–19]. The smaller number, longer course, and flatter, collapsible nature of CCs in human eyes may increase the risk of outflow failure and IOP elevation in glaucoma. Consistent with this, Hann et al. described 3.7 times more CC occlusions in eyes with glaucoma [37]. It will be interesting to compare the outflow vessel glycocalyx pattern in RSCM scans of eyes with glaucoma to look for clues of an altered wall adherence.

In the ST_q_ of eye 2, there was one region with a weak signal and low volumes that corresponded to the corneoscleral tunnel created during cataract surgery while the adjacent ones were enlarged. Considering that eye 2 had surgery and no history of glaucoma, it is tempting to speculate that the opening CSAs or the remaining adjacent vessels enlarged to facilitate flow. This matches an increased count of CCs at higher IOPs as reported by Hann et al. [37].

In the anterior segment, the long ciliary nerve has prominent branches of unknown function, termed Axenfeld loops [23]. Reese described them as a retroverted nerve loop positioned on the scleral surface, with occasionally accompanying cyst-like structures that are more prominent in glaucoma [24]. With our present clearing protocol, we could not perform immunohistology on cleared specimens to confirm that these characteristic structures that were present in all quadrants were in fact nerve loops. Their frequent presence at regular intervals in proximity and parallel to CCs suggests that they might play a role related to the innervation of TM, scleral spur [38], collector channels or EVP [39] to participate in the autonomous regulation of outflow [40].

Our study had several limitations. A more comprehensive analysis with additional eyes would have been desirable, but the relative scarcity of human donor eyes and the intense computational demand required a focused approach. The experiments were not meant to measure or compare outflow function Such structure-function correlation or computational fluidics simulation studies will be necessary in the future. We did not design or power this study to detect potential differences caused by gender or lens status. It is possible that lens status and gender contributed to interindividual differences even if these variables were not found to impact outflow in the anterior segment perfusion model [41–43] we normally use [44,45]. A recent study by Strenk et al. [46] demonstrated that vector forces on the outflow system change in pseudophakic eyes in a way that can impact outflow system geometry which might have occurred here as well. Any potential posterior displacement of lens and iris caused by the dye bolus and 90-minute dye perfusion would have increased conventional outflow helping the label to reach vascular structures participating in outflow in this model [47]. Any effect from variable fluid preloading would be more relevant in outflow studies with IOPs below 20 mmHg [48].

Additionally, some locations of artificial discontinuity were seen in CC surfaces generated by Imaris. When viewed in the original fluorescence data, all CCs could be manually traced from points of connection from the SC to the SVP. While Imaris surfaces created informative approximations of outflow structures, this automated segmentation may need to be supplemented with manual segmentation for data that might be used in future fluid dynamics studies.

In summary, we analyzed the human conventional outflow tract of octogenarian eyes in full-thickness, high-resolution and 3D. Using their CC glycosaminoglycan lectin staining profile, we characterized CSA, length, and branching patterns. Key differences between the human and porcine outflow tract may help explain why humans—but not pigs—frequently develop glaucoma. Future studies will need to assess glaucoma specific changes of morphology and glycocalyx composition of the conventional outflow tract.

## Supporting information

S1 Fig A) Human and porcine CC CSA (left) and representative images of measured CC openings (top: human, bottom: porcine). B) Human and porcine CC area coverage (left) and images of CC opening areas as shown in A. C) Human and porcine CC length (left) and representative CCs traced (orange). CC: collector channel, CSA: cross-section area.
(TIFF)

S1 Movie Fly-through movie of eye 1. (MP4)

S2 Movie Fly-through movie of the supranasal quadrant of eye 1. (MP4)

S1 Table Surface parameter ranges. (DOCX)
