# Supplementary figures and images for: 3D-Reconstruction of the human conventional outflow system by ribbon scanning confocal microscopy
Source: PLoS One. 2020 May 18;15(5):e0232833. doi: 10.1371/journal.pone.0232833 (PMC7233539; doi:10.1371/journal.pone.0232833)

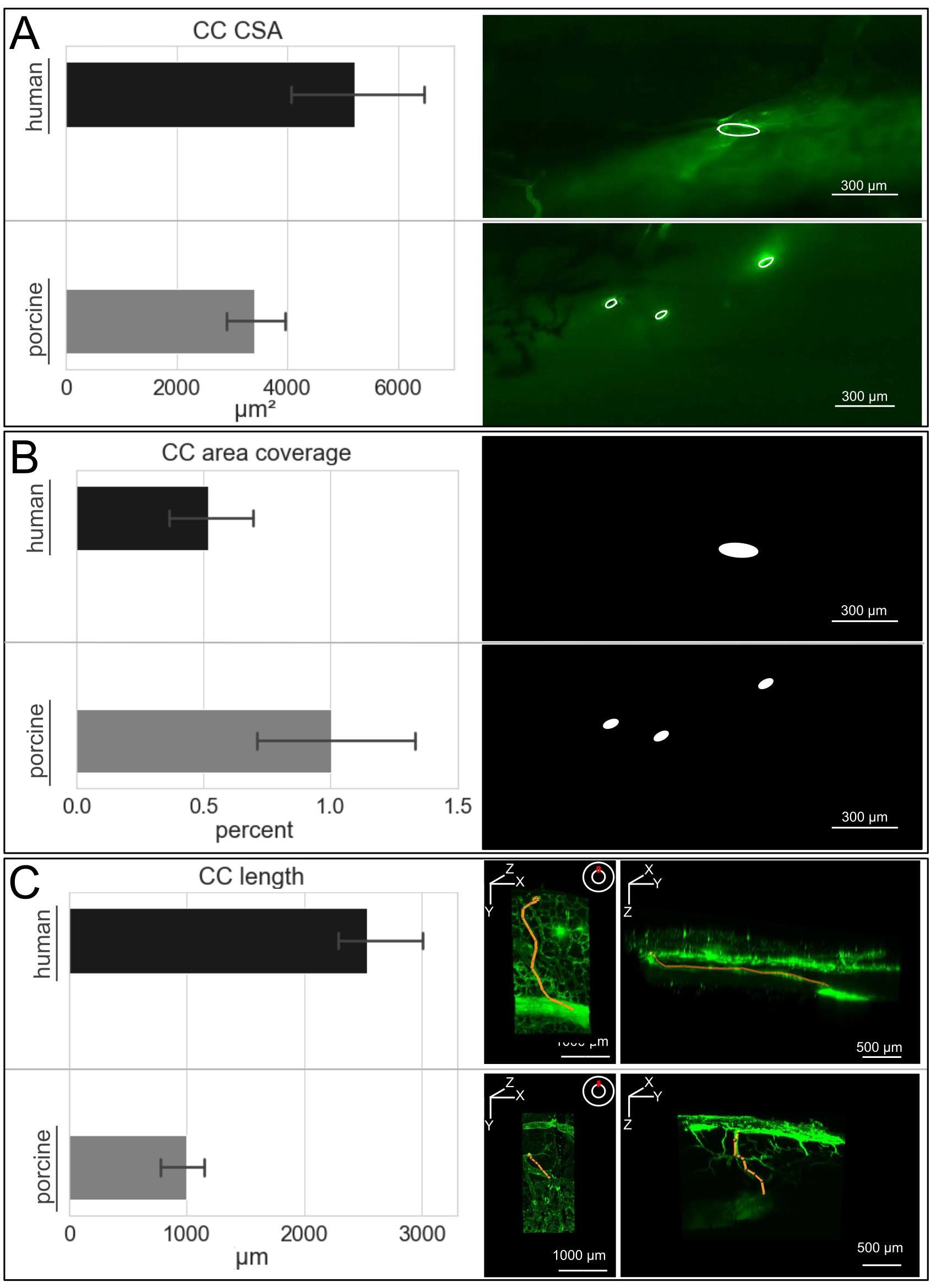

Supplement: S1 Fig — A) Human and porcine CC CSA (left) and representative images of measured CC openings (top: human, bottom: porcine). B) Human and porcine CC area coverage (left) and images of CC opening areas as shown in A. C) Human and porcine CC length (left) and representative CCs traced (orange). CC: collector channel, CSA: cross-section area. (TIFF) [file pone.0232833.s001.tiff]
