# Supplementary material for: 3D-Reconstruction of the human conventional outflow system by ribbon scanning confocal microscopy
Source: PLoS One. 2020 May 18;15(5):e0232833. doi: 10.1371/journal.pone.0232833 (PMC7233539; doi:10.1371/journal.pone.0232833)
Supplement: S1 Table — (DOCX) [file pone.0232833.s004.docx]

## Supplemental table 1

### Surface Parameter Ranges

|  | surface grain size (µm) | background subtraction- largest sphere diameter (µm) | background subtraction-  Threshold Value | background subtraction- Threshold Value B | Filter  “Number of Voxels” |
| --- | --- | --- | --- | --- | --- |
| SVP  (Eye 1) | 3.00 | 45.0-200 | 600-1387 | 28444.1-51500 | >1.00e5-4.79e6 |
| SVP  (Eye 2) | 3.00 | 200 | 691 | 48422.7-57016.4 | >2.53e6-3.04e8 |
| SVP/CC  (Eye 1) | 3.00 | 10.0 -90.0 | 600-1200 | 51500-57487.6 | >1.00e4-4.79e6 |
| SVP/CC  (Eye 2) | 3.00 | 200 | 691 | 27340.5-57774.6 | >10.0-4.23e8 |
| SVP/CC  (Eye 1) | 3.00 | 10.0 -90.0 | 600-1200 | 51500-57487.6 | >1.00e4-4.79e6 |
| TM  (Eye 1) | 2.60-3.00 | 10.0-15.0 | 480-1200 | 16381.5-29148.4 | >1.00-1.00e4 |
| TM  (Eye 2) | 3.00 | 200 | 691 | 16062.4-43850.6 | >10.0-1.16e8 |

### Eye 1

| **SVP** | surface grain size | background subtraction- largest sphere diameter | background subtraction-  Threshold Value | background subtraction- Threshold Value B | Filter  “Number of Voxels” |
| --- | --- | --- | --- | --- | --- |
| Q1, Surface 85 | 3.00 µm | 45.0 µm | 1384 | 51500 | **>**4.79e6 |
| Q2, Surface 31 | 3.00 µm | 90.0 µm | 600 | 41065 |  |
| Q3, Surface 30 | 3.00 µm | 80.0 µm | 1200 | 28444.1 | **>**1.00e5 |
| Q4, Surface 84 | 3.00 µm | 200 µm | 1387 | 51500 | **>**7.77e5 |
| Eye 1 SVP Range | 3.00 µm | 45.0-200 µm | 600-1387 | 28444.1-51500 | >1.00e5-4.79e6 |

| **SVP/CC** | surface grain size | background subtraction- largest sphere diameter | background subtraction-  Threshold Value | background subtraction- Threshold Value B | Filter  “Number of Voxels” |
| --- | --- | --- | --- | --- | --- |
| Q1, Surface 88 | 3.00 µm | 10.0 µm | 600 | 51500 | >4.79e6 |
| Q2, Surface 27 | 3.00 µm | 90.0 µm | 640 | 53823.9 | >1.00e4 |
| Q3, Surface 46 | 3.00 µm | 90.0 µm | 1200 | 57487.6 | >1.00e5 |
| Q4, Surface 42 | 3.00 µm | 20.0 µm | 600 | 51500 | >1.00e4 |
| Eye 1 SVP/CC Range | 3.00 µm | 10.0 -90.0 µm | 600-1200 | 51500-57487.6 | >1.00e4-4.79e6 |

| **TM** | surface grain size | background subtraction- largest sphere diameter | background subtraction-  Threshold Value | background subtraction- Threshold Value B | Filter  “Number of Voxels” |
| --- | --- | --- | --- | --- | --- |
| Q1, Surface 10 | 2.60 µm | 15.0 µm | 600 | 29148.4 |  |
| Q2, Surface 7 | 3.00 µm | 10.0 µm | 1200 | 16381.5 | >1.00 |
| Q3, Surface 11 | 2.60 µm | 15.0 µm | 480 | 20511 |  |
| Q4, Surface 53 | 2.60 µm | 15.0 µm | 680 | 26638.7 | >1.00e4 |
| Eye 1 TM Range | 2.60-3.00 µm | 10.0-15.0 µm | 480-1200 | 16381.5-29148.4 | >1.00-1.00e4 |

### Eye 2

| **SVP** | surface grain size | background subtraction- largest sphere diameter | background subtraction-  Threshold Value | background subtraction- Threshold Value B | Filter  “Number of Voxels” |
| --- | --- | --- | --- | --- | --- |
| Q4, Surface 13 | 3.00 µm | 200 µm | 691 | 48422.7 | > 3.48e7 |
| Q3, Surface 5 | 3.00 µm | 200 µm | 691 | 57016.4 | > 2.53e6 |
| Q2, Surface 29 | 3.00 µm | 200 µm | 691 | 51045.5 | > 4.44e6 |
| Q1, Surface 26 | 3.00 µm | 200 µm | 691 | 52597.5 | > 3.04e8 |
| Eye 2  SVP Range | 3.00 µm | 200 µm | 691 | 48422.7-57016.4 | >2.53e6-3.04e8 |

| **SVP/CC** | surface grain size | background subtraction- largest sphere diameter | background subtraction-  Threshold Value | background subtraction- Threshold Value B | Filter  “Number of Voxels” |
| --- | --- | --- | --- | --- | --- |
| Q4, Surface 15 | 3.00 µm | 200 µm | 691 | 57774.6 | >4.77e6 |
| Q4, Surface 14 | 3.00 µm | 200 µm | 691 | 35586.6 | >4.73e7 |
| Q3, Surface 13 | 3.00 µm | 200 µm | 691 | 27340.5 | >10.0 |
| Q1, Surface 25 | 3.00 µm | 200 µm | 691 | 50990.8 | >2.59e6  <4.23e8 |
| Eye 2 SVP/CC Range | 3.00 µm | 200 µm | 691 | 27340.5-57774.6 | >10.0-4.23e8 |

| **TM** | surface grain size | background subtraction- largest sphere diameter | background subtraction-  Threshold Value | background subtraction- Threshold Value B | Filter  “Number of Voxels” |
| --- | --- | --- | --- | --- | --- |
| Q4, Surface 2 | 3.00 µm | 200 µm | 691 | 36092.5 | >5.22e6 |
| Q4, Surface 11 | 3.00 µm | 200 µm | 691 | 16062.4 | >7.74e7 |
| Q2, Surface 7 | 3.00 µm | 200 µm | 691 | 43850.6 | >2.20e7  <1.16e8 |
| Q1, Surface 14 | 3.00 µm | 200 µm | 691 | 22287 | >10.0 |
| Eye 2 TM Range | 3.00 µm | 200 µm | 691 | 16062.4-43850.6 | >10.0-1.16e8 |

### Eye 1:

### SVP:

-Eye 1, Quadrant 1, Surface 85

Surface Grain Size = 3.00 µm

Enable Eliminate Background = true

Diameter Of Largest Sphere = 45.0 µm

[Threshold]

Enable Automatic Threshold = false

Manual Threshold Value = 1384

Active Threshold = true

Enable Automatic Threshold B = false

Manual Threshold Value B = 51500

Active Threshold B = true

[Classify Surfaces]

"Number of Voxels Img=1" above 4.79e6

-Eye 1, Quadrant 2, Surface 31

Surface Grain Size = 3.00 µm

Enable Eliminate Background = true

Diameter Of Largest Sphere = 90.0 µm

[Threshold]

Enable Automatic Threshold = false

Manual Threshold Value = 600

Active Threshold = true

Enable Automatic Threshold B = true

Manual Threshold Value B = 41065

Active Threshold B = false

[Classify Surfaces]

"Volume" above 1.00e4 µm^3

-Eye 1, Quadrant 3, Surface 30

Surface Grain Size = 3.00 µm

Enable Eliminate Background = true

Diameter Of Largest Sphere = 80.0 µm

[Threshold]

Enable Automatic Threshold = false

Manual Threshold Value = 1200

Active Threshold = true

Enable Automatic Threshold B = false

Manual Threshold Value B = 28444.1

Active Threshold B = false

[Classify Surfaces]

"Number of Voxels Img=1" above 1.00e5

-Eye 1, Quadrant 4, Surface 84

Surface Grain Size = 3.00 µm

Enable Eliminate Background = true

Diameter Of Largest Sphere = 200 µm

[Threshold]

Enable Automatic Threshold = false

Manual Threshold Value = 1387

Active Threshold = true

Enable Automatic Threshold B = false

Manual Threshold Value B = 51500

Active Threshold B = true

[Classify Surfaces]

"Number of Voxels Img=1" above 7.77e5

### SVP/CC:

-Eye 1, Quadrant 1, Surface 88

Surface Grain Size = 3.00 µm

Enable Eliminate Background = true

Diameter Of Largest Sphere = 10.0 µm

[Threshold]

Enable Automatic Threshold = false

Manual Threshold Value = 600

Active Threshold = true

Enable Automatic Threshold B = false

Manual Threshold Value B = 51500

Active Threshold B = true

[Classify Surfaces]

"Number of Voxels Img=1" above 4.79e6

-Eye 1, Quadrant 2, Surface 27

Surface Grain Size = 3.00 µm

Enable Eliminate Background = true

Diameter Of Largest Sphere = 90.0 µm

[Threshold]

Enable Automatic Threshold = false

Manual Threshold Value = 640

Active Threshold = true

Enable Automatic Threshold B = true

Manual Threshold Value B = 53823.9

Active Threshold B = false

[Classify Surfaces]

"Number of Voxels Img=1" above 1.00e4

-Eye 1, Quadrant 3, Surface 46

Surface Grain Size = 3.00 µm

Enable Eliminate Background = true

Diameter Of Largest Sphere = 90.0 µm

[Threshold]

Enable Automatic Threshold = false

Manual Threshold Value = 1200

Active Threshold = true

Enable Automatic Threshold B = false

Manual Threshold Value B = 57487.6

Active Threshold B = false

[Classify Surfaces]

"Number of Voxels Img=1" above 1.00e5

-Eye 1, Quadrant 4, Surface 42

Surface Grain Size = 3.00 µm

Enable Eliminate Background = true

Diameter Of Largest Sphere = 20.0 µm

[Threshold]

Enable Automatic Threshold = false

Manual Threshold Value = 600

Active Threshold = true

Enable Automatic Threshold B = false

Manual Threshold Value B = 51500

Active Threshold B = true

[Classify Surfaces]

"Number of Voxels Img=1" above 1.00e4

### TM:

-Eye 1, Quadrant 1, Surface 10

Surface Grain Size = 2.60 µm

Enable Eliminate Background = true

Diameter Of Largest Sphere = 15.0 µm

[Threshold]

Enable Automatic Threshold = false

Manual Threshold Value = 600

Active Threshold = true

Enable Automatic Threshold B = false

Manual Threshold Value B = 29148.4

Active Threshold B = false

[Classify Surfaces]

"Volume" above 1.00e4 µm^3

-Eye 1, Quadrant 2, Surface 7

Surface Grain Size = 3.00 µm

Enable Eliminate Background = true

Diameter Of Largest Sphere = 10.0 µm

[Threshold]

Enable Automatic Threshold = false

Manual Threshold Value = 1200

Active Threshold = true

Enable Automatic Threshold B = true

Manual Threshold Value B = 16381.5

Active Threshold B = false

[Classify Surfaces]

"Number of Voxels Img=1" above 1.00

-Eye 1, Quadrant 3, Surface 11

Surface Grain Size = 2.60 µm

Enable Eliminate Background = true

Diameter Of Largest Sphere = 15.0 µm

[Threshold]

Enable Automatic Threshold = false

Manual Threshold Value = 480

Active Threshold = true

Enable Automatic Threshold B = false

Manual Threshold Value B = 20511

Active Threshold B = false

[Classify Surfaces]

"Volume" above 1.00e4 µm^3

-Eye 1, Quadrant 4, Surface 53

Surface Grain Size = 2.60 µm

Enable Eliminate Background = true

Diameter Of Largest Sphere = 15.0 µm

[Threshold]

Enable Automatic Threshold = false

Manual Threshold Value = 680

Active Threshold = true

Enable Automatic Threshold B = false

Manual Threshold Value B = 26638.7

Active Threshold B = false

[Classify Surfaces]

"Number of Voxels Img=1" above 1.00e4

### Eye 2:

### SVP:

-Eye 2, Quadrant 4, Surface 13

Surface Grain Size = 3.00 µm

Enable Eliminate Background = true

Diameter Of Largest Sphere = 200 µm

[Threshold]

Enable Automatic Threshold = false

Manual Threshold Value = 691

Active Threshold = true

Enable Automatic Threshold B = true

Manual Threshold Value B = 48422.7

Active Threshold B = false

[Classify Surfaces]

"Number of Voxels" above 3.48e7

-Eye 2, Quadrant 3, Surface 5

Surface Grain Size = 3.00 µm

Enable Eliminate Background = true

Diameter Of Largest Sphere = 200 µm

[Threshold]

Enable Automatic Threshold = false

Manual Threshold Value = 691

Active Threshold = true

Enable Automatic Threshold B = true

Manual Threshold Value B = 57016.4

Active Threshold B = false

[Classify Surfaces]

"Number of Voxels" above 2.53e6

-Eye 2, Quadrant 2, Surface 29

Surface Grain Size = 3.00 µm

Enable Eliminate Background = true

Diameter Of Largest Sphere = 200 µm

[Threshold]

Enable Automatic Threshold = false

Manual Threshold Value = 691

Active Threshold = true

Enable Automatic Threshold B = true

Manual Threshold Value B = 51045.5

Active Threshold B = false

[Classify Surfaces]

"Number of Voxels" above 4.44e6

-Eye 2, Quadrant 1, Surface 26

Surface Grain Size = 3.00 µm

Enable Eliminate Background = true

Diameter Of Largest Sphere = 200 µm

[Threshold]

Enable Automatic Threshold = false

Manual Threshold Value = 691

Active Threshold = true

Enable Automatic Threshold B = true

Manual Threshold Value B = 52597.5

Active Threshold B = false

[Classify Surfaces]

"Number of Voxels" above 3.04e8

### SVP/CC:

-Eye 2, Quadrant 4, Surface 15

Surface Grain Size = 3.00 µm

Enable Eliminate Background = true

Diameter Of Largest Sphere = 200 µm

[Threshold]

Enable Automatic Threshold = false

Manual Threshold Value = 691

Active Threshold = true

Enable Automatic Threshold B = true

Manual Threshold Value B = 57774.6

Active Threshold B = false

[Classify Surfaces]

"Number of Voxels" above 4.77e6

-Eye 2, Quadrant 4, Surface 14

Surface Grain Size = 3.00 µm

Enable Eliminate Background = true

Diameter Of Largest Sphere = 200 µm

[Threshold]

Enable Automatic Threshold = false

Manual Threshold Value = 691

Active Threshold = true

Enable Automatic Threshold B = true

Manual Threshold Value B = 35586.6

Active Threshold B = false

[Classify Surfaces]

"Number of Voxels" above 4.73e7

-Eye 2, Quadrant 3, Surface 13

Surface Grain Size = 3.00 µm

Enable Eliminate Background = true

Diameter Of Largest Sphere = 200 µm

[Threshold]

Enable Automatic Threshold = false

Manual Threshold Value = 691

Active Threshold = true

Enable Automatic Threshold B = true

Manual Threshold Value B = 27340.5

Active Threshold B = false

[Classify Surfaces]

"Number of Voxels" above 10.0

-Eye 2, Quadrant 1, Surface 25

Surface Grain Size = 3.00 µm

Enable Eliminate Background = true

Diameter Of Largest Sphere = 200 µm

[Threshold]

Enable Automatic Threshold = false

Manual Threshold Value = 691

Active Threshold = true

Enable Automatic Threshold B = true

Manual Threshold Value B = 50990.8

Active Threshold B = false

[Classify Surfaces]

"Number of Voxels" between 2.59e6 and 4.23e8

### TM:

-Eye 2, Quadrant 4, Surface 2

Surface Grain Size = 3.00 µm

Enable Eliminate Background = true

Diameter Of Largest Sphere = 200 µm

[Threshold]

Enable Automatic Threshold = false

Manual Threshold Value = 691

Active Threshold = true

Enable Automatic Threshold B = true

Manual Threshold Value B = 36092.5

Active Threshold B = false

[Classify Surfaces]

"Number of Voxels" above 5.22e6

-Eye 2, Quadrant 4, Surface 11

Surface Grain Size = 3.00 µm

Enable Eliminate Background = true

Diameter Of Largest Sphere = 200 µm

[Threshold]

Enable Automatic Threshold = false

Manual Threshold Value = 691

Active Threshold = true

Enable Automatic Threshold B = true

Manual Threshold Value B = 16062.4

Active Threshold B = false

[Classify Surfaces]

"Number of Voxels" above 7.74e7

-Eye 2, Quadrant 2, Surface 7

Surface Grain Size = 3.00 µm

Enable Eliminate Background = true

Diameter Of Largest Sphere = 200 µm

[Threshold]

Enable Automatic Threshold = false

Manual Threshold Value = 691

Active Threshold = true

Enable Automatic Threshold B = true

Manual Threshold Value B = 43850.6

Active Threshold B = false

[Classify Surfaces]

"Number of Voxels" between 2.20e7 and 1.16e8

-Eye 2, Quadrant 1, Surface 14

Surface Grain Size = 3.00 µm

Enable Eliminate Background = true

Diameter Of Largest Sphere = 200 µm

[Threshold]

Enable Automatic Threshold = false

Manual Threshold Value = 691

Active Threshold = true

Enable Automatic Threshold B = true

Manual Threshold Value B = 22287

Active Threshold B = false

[Classify Surfaces]

"Number of Voxels" above 10.0
